# Type 1 diabetes mellitus and gluten induced disorders 

**Published:** 2014

**Authors:** Sabine Hogg-Kollars, David Al Dulaimi, Karen Tait, Kamran Rostami

**Affiliations:** 1 *Institute of Health & Society, University of Worcester, UK*; 2 *Department of Gastroenterology, Alexandra Hospital, Redditch, UK*; 3 *Department of General Medicine, Alexandra Hospital, Redditch, UK *

**Keywords:** Coeliac disease, Genes, Gluten free diet, Malabsorption, Type 1 diabetes mellitus

## Abstract

Over the last five decades the association between coeliac disease and other autoimmune disorders such as autoimmune thyroid disease or diabetes mellitus type 1 has been well established through many studies and to this day is subject to on-going clinical and scientific investigation worldwide. While no link has been established between celiac disease and type-2 diabetes mellitus, coeliac disease is common in patients with type 1 diabetes. The improvement of symptoms in patients with both conditions through dietary intervention, in the form of a gluten free diet, has been widely described within the literature. Our objectives were to review and synthesise the current knowledge on the nutritional treatment for patients with both conditions.

## Introduction

 Coeliac Disease (CD), also referred to as gluten-sensitive enteropathy, is an autoimmune condition that occurs in genetically predisposed people by exposure to gluten. Initially mentioned in literature in the second century AD, CD was described as an intestinal disorder with symptoms of diarrhoea, malabsorption and weight loss. Recently, it has become clear that there is a group of conditions related to gluten consumption. Foremost among them are three types: a) the least common is wheat allergy; b) the autoimmune form, the best characterized, includes CD, dermatitis herpetiformis, and gluten ataxia; and c) sensitivity to gluten, which is possibly immune-mediated and now the most common (1). An association between gluten and CD was only established much later, by Dicke (1953), who found that the removal of gluten from patients’ diets led to the improvement of symptoms (2). 

**Gluten and gluten toxicity**

Gluten is a protein constituent found in wheat, rye, and barley. It is gluten that gives dough its elasticity, helps it rise and contributes to the texture of many food products such as bread, pasta, or imitation meats (3,4). Specifically the storage proteins (prolamins) gliadin (wheat), secalin (rye), and hordein (barley) have been shown to have toxic effects on intestinal cells in gluten sensitive people. The toxic effects of these prolamins include the reduction of F-actin, inhibition of cellular growth, premature cell death, the rearrangement of the cytoskeleton, and increased small bowel permeability (5).

**Symptoms and associations of CD**

Despite a strong association with classic symptoms such as abdominal pain, chronic diarrhoea, and vomiting; symptoms such as faltering growth in childhood, anaemia, extreme fatigue, and tiredness are also very common in CD. The condition can also affect the musculoskeletal, neurological, and reproductive system, but may remain completely asymptomatic in some subjects (6). The symptoms of CD are accompanied by chronic inflammation of the lamina propria, an increase in intraepithelial lymphocytes and small bowel villous atrophy (6,7). The inflammation affecting the intestinal villi compromises the absorptive capacity of the small intestine. This is associated with impaired nutrient absorption and nutritional deficiencies. Low serum levels of folate, vitamin B12, iron, zinc, copper, calcium, and magnesium are commonly seen in patients who suffer from CD (8,9).

CD is considered to result from an interaction between environmental factors, a genetic predisposition and immune mediated intolerance to gliadin. There is evidence of environmental triggers other than gluten linked to the pathogenesis of this condition. A number of studies suggest a causative role of viruses, specifically the Adenovirus type 12, in the disease development via a mechanism referred to as molecular mimicry in which gliadin shares an amino acid sequence with the virus. Other studies have failed to establish such a link (10). Mahon et al. (1991) investigated the presence of persisting Adenovirus 12 infection in the small intestinal mucosa of 18 CD patients. The researchers found a low prevalence of the infection (n=4) among CD patients and stated that the incidence of Adenovirus 12 infection may not correlate with the incidence of CD (11). The impact of the lack of breastfeeding and exposure to gluten in early infancy has been widely discussed within literature (12). 

**Epidemiology**

CD can develop at any age. The majority of patients with CD now present with relatively mild symptoms and indeed over a third of patients diagnosed in this population have few if any symptoms (13). It is one of the most common chronic disorders with a large variability in regards to its prevalence worldwide (14). While European and US studies highlight a prevalence rate of roughly 1% within the general population, CD appears to be more prevalent among patients affected by type 1 diabetes (T1DM), with up to 16% found amongst child T1DM sufferers (15,16). A higher prevalence of CD among patients with T1DM was also reported by a more recent North Indian study; Bhadada and co-workers demonstrated a CD prevalence of over 11% among patients with T1DM (17).

 Cerutti et al. in their study from 2004 highlighted the impact of gender and age on the risk of developing CD and T1DM. The researchers concluded that risk of developing both conditions increased with female gender and with a diagnosis of T1DM at age <4 years. People that suffer from both conditions, T1DM and CD, are thought to develop T1DM earlier in life than people who are affected by T1DM only (18). As with CD, patients with T1DM are prone to also suffer from other autoimmune conditions. In this context, Not et al. found that the prevalence of other autoimmune conditions was significantly higher in people who suffered from both conditions, CD and T1DM (19). 

**CD linked to T1DM**

The association between CD and T1DM was first reported in the late 1960s by Walker-Smith at al. who described symptoms of CD in children diagnosed with T1DM (20), a metabolic disorder which accounts for 5-10% of all diabetes cases (16) and affects roughly 0.4% of persons of European origin (21,22). T1DM usually develops during childhood and young adulthood. In T1DM abnormal activation of the immune system leads to an inflammatory response within the pancreatic islets and to a β-cell response involving the production of antibodies to β-cells resulting in their destruction of β-cells followed by absolute insulin deficiency and hyperglycaemia (23). Environmental factors such as viral infections, toxins such as nitrosamines and early exposure to milk protein and gluten are thought to be linked to the pathogenesis of this condition in the genetically predisposed (24,25).

The susceptibility to develop both conditions appears to be largely inherited; CD and T1DM share common genetic make-up with the HLA-DQB1 gene on chromosome *6p21* being affected in the majority of patients who suffer from both conditions. Over 90% of CD cases and roughly 70% of patients with T1DM are thought to carry the HLA heterodimer DQA1*05DQb1*0201; HLA genes are essential to the regulation of the immune response (26,27). Many of the HLA-DQ loci have been found in association with other autoimmune diseases such as Hashimototo´s thyroiditis and rheumatoid arthritis (28). 

**Diagnosis**

The diagnosis of CD involves serological screening tests for tissue transglutaminase (TTG), endomysial antibodies (EMA) and deaminated gliadin peptides (IgA and IgG DGP). In order to confirm villous atrophy, small intestinal biopsy from duodenum, the current gold standard of diagnosis, is recommended. Although mostly undetected, the majority of CD cases are thought to be already present at the onset of diabetes (13). When the two conditions concur, serum markers for CD (IgA antibodies to tissue transglutaminase and endomysium) have been found to be present in 60% of T1DM patients (who are going to develop CD in the future) at diabetes onset; Barera et al. (2002) also reported that an estimated 40% of subjects suffering from T1DM develop CD after diabetes onset (13). Potential false-negative results may occur in cases with IgA deficiency typically seen in 2-3% patients with CD. In these cases, testing for IgG autoantibodies is recommended (29). 

**Screening**

Suffering from two long-term conditions, such as CD and T1DM, can have a severe impact on quality of life for patients (17). Untreated CD is associated with an increased risk of developing small bowel cancer or lymphoma (12). Both conditions are linked to osteoporosis, a condition characterised by increased risk of bone fracture (30). Furthermore, CD undetected in T1DM patients is associated with a higher prevalence of retinopathy, nephropathy and poor glycaemic control (31). It appears that most patients suffering from both CD and T1DM conditions do not present with typical gastro-intestinal symptoms (28); CD is often subclinical, asymptomatic or may present with atypical features such as fertility problems, osteopenia, hepatic steatosis, ataxia, epilepsy, and alopecia (28). For these reasons, some clinicians have advocated interval screening for CD in T1DM patients.

Although CD is highly prevalent among T1DM patients, there is currently no international consensus on the screening. National Institute for Health and Clinical Excellence (NICE) recommends CD screening at diagnosis of T1DM even in the absence of clinical CD symptoms, other organisations suggest that screening should be performed only in the presence of symptoms (32). The recommendation is for active case finding but not mass screening (33). A variation in practice is supported by a most recent survey from North America that demonstrated a lack of uniformity at present. Apart from a low screening frequency, Simpson et al. (2013) also highlighted inconsistency in the management of CD and a lack of education regarding the treatment of patients with both conditions amongst dieticians (22). 

**Gluten free diet (GFD) and glycaemic index (GI) diet**

The management of T1DM requires a lifelong diet and insulin therapy. T1DM is treated with insulin replacement not dietary exclusion however bolus insulin dose adjustment for carbohydrate portions and an awareness of glycaemic index (GI) are both important in achieving stable glycaemia. This is recognised in guidelines by NICE (2010) and the American Association of Clinical Endocrinologists (2010), which recommended a low glycaemic diet as dietary treatment. A low GI diet has a beneficial effect on post-prandial hyperglycaemia, reduces the risk of developing cardiovascular disease and the associated premature mortality in individuals with this condition (34,35). The concept of glycaemic index (GI) is a modern concept that was first introduced in the 1980s (36). It involves the scoring of carbohydrate containing foods depending on their effect on postprandial glycaemia, in the hope of optimising glycaemic control. Strict glycaemic control in T1DM patients can reduce the likelihood of developing complications associated with T1DM and can improve patients’ life quality (37). Known barriers to strict glycaemic control include ability to estimate the GI of a meal, access to low GI foods and limited choice of food. The role of the GI education for health personnel and patients has been highlighted (36). It is challenging for patients to consider GI, carbohydrate portions and then gluten exclusion when CD is diagnosed. 

When positive serum markers and small bowel biopsy confirm CD, a gluten free diet (GFD) is initiated. A GFD involves the total exclusion of all gluten including wheat, rye and barley and gluten contaminated foods as can be the case with oats; even small amounts of gluten is known to provoke immune mediated response (38). Wheat starch has been used as part of a gluten free diet. To meet the standards for gluten free it is industrially purified according to Codex standards. The maximal amount of protein allowed in wheat starch is 0.3% in the dry matter (39). As highlighted by studies from Sweden, Finland and the UK, the availability of gluten free products increases regarding the complete recovery of the intestinal mucosa (40,41). Although wheat starch-derived gluten-free products may still carry gluten in minimal amounts, a randomised study comparing wheat starch-based gluten-free products to naturally gluten-free products found no differences concerning morphological and clinical responses (42). Although the permanent removal of gluten from the diet is essential for maintenance of a normal intestinal mucosa, it is questionable if a strict GFD is nutritionally balanced (43). Hallert et al. (2002) investigated the nutrient status of CD subjects with biopsy proven remission after an 8-12 year GFD. The researchers found that these subjects showed lower plasma level of folate and pyridoxal-5´-phospate than controls. The mean daily intake of folate and vitamin B12 was also significantly lower than that of controls from the general population with the same age (44).

Histological responses to the withdrawal of gluten are variable. The recovery of the intestinal mucosa can take months to years once the GFD has been established (40). Ciacci et al. (2002) showed in a cohort study involving 390 CD subjects on a GFD that adherence to a strict GFD has a significant impact on the intestinal damage in CD patients (45).

However, there is conflicting evidence on the effects and benefit of a gluten free diet on diabetes symptoms for patients with both conditions. A recent study from 2011 by Sherif et al. found no link between a gluten free diet and glycaemic control or growth in children affected by both T1DM and CD (46). Studies not in support of an effect in T1DM were either disrupted by poor compliance to a GFD (47,48), were confounded by lack of control subjects (20,48-53) or used inadequate longitudinal follow-up data (47,50,53,54). 

According to works by Rubin & Peyrot (1999) and Nachman et al. (2009) symptom improvements only become apparent after 1 year of total gluten exclusion (55,56). A small longitudinal study by Amin et al. (2002) investigating parameters before and after the administration of a gluten free diet found a recovery of glycated haemoglobin (HbA1c) and BMI SDS in people who followed a GFD with no change in insulin dose (57). In a more recent study by Leeds et al. (2011) adults with undetected CD and T1DM were found to have worse glycaemic control and a higher prevalence of retinopathy and nephropathy than their controls. The researchers found that patients complying with a GFD showed a significant increase in HDL cholesterol, improvements in glycaemic control as well as renal protein excretion and modest improvements in HbA1c. Leeds et al. (2011) concluded that in their study GFD did not show a negative impact on life quality (31). 

There are other studies examining the beneficial association between a GFD and T1DM. A recent study from 2009 found that 1-year of a GFD increased the HDL cholesterol levels by 18% without changing total cholesterol levels (58). In addition, an Italian study investigating the bone metabolism in the presence of both conditions found that the quality of bone in patients with T1DM and CD on a strictly GFD was similar to the quality found in patients with T1DM only. The researchers also found that osteopenia was more prevalent in patients with both diseases who reported habitual transgressions to a GFD (59). 

Further evidence for the beneficial effects of a GFD stems from a study by Malalasekera et al. (2009). The researchers compared children with CD and T1DM on a GFD with a control group of diabetes subjects with T1DM only. Children with CD showed a lower urinary protein loss than their controls, which may suggest a protective value of a GFD (60). 

**Adherence**

Adherence to a GFD is associated with the improvement of symptoms of both conditions; it appears to have a protective effect (61-63). In cases where CD is asymptomatic, the main reason for the introduction of a gluten free diet is preventing nutritional deficiencies and reduce the risk of long term complications like autoimmune disorders and malignancies related to celiac disease (61,64). However, few studies have been carried out to determine compliance in asymptomatic patients. In this context a recent study by Chauhan et al. (2010) found that 18% of children affected by CD did not comply (65). In a Swedish study from 2003 age at diagnosis of CD is discussed as a factor; only 36% of adults who had been diagnosed after the age of 4 adhered to a gluten free diet where as 80% of adults who had been diagnosed before the age of 4 complied (66). Non-compliance in CD is multifactorial. Factors associated with poor compliance include lack of family support, lack of access to gluten free foods and limited knowledge on the harmful effects of gluten in CD patients. Other adverse factors include affordability of a GFD, with gluten free foods being more expensive than their gluten containing counterparts (67-69) and insufficient food labelling (70). As GFD and gluten-free products can be deficient in vitamins and minerals such as vitamin D, B vitamins, calcium, zinc, iron, magnesium, and fiber, the administration of nutritional supplements appears common practice. Supplementation has been found to help malabsorption and co-morbidities related to nutritional deficiencies provided that supplements do not contain traces of starches, malt, malt syrup, dextrin, alcohol, and flours deriving from gluten grains (43). There is evidence that people with diabetes feel challenged by their condition and its management via diet and insulin administration. Patients' quality of life can potentially improve by reducing episodes of hyperglycaemia or can worsen by increasing episodes of hypoglycaemia. The psychological burden in diabetes is substantial. When a patient is confronted with both conditions, CD and T1DM, psychosocial factors such as depression have a profound effect upon treatment outcome (71,72). Emerging alternatives to a GFD are enzyme therapies. These involve the administration of endoprotease and endopeptidases to food, inhibitors of intestinal permeability and DQ2 peptide blockers (73-75). 

It seems intuitive that in cases where both autoimmune conditions are present, a well-educated nutrition expert should be an integral part of the health care team in order to monitor the diet and avoid or correct nutritional inadequacies (36,43). In this context a recent study from 2013 on the effect of dietician use on the outcome of coeliac disease could not establish a link between parameters such as dietary adherence, life quality or the severity of symptoms and dietician use. A study by Ukkola et al. also failed to demonstrate a correlation between patients´ knowledge and dietician follow-up, but showed that patients often requested more detailed dietary counselling, suggesting insufficient dietician contact (76). An annual review, in a dietician led clinic, can significantly improve compliance - as demonstrated in a cohort study by Wylie et al. (2005) (77).

## Conclusion

CD and T1DM are two chronic conditions that often occur together in the same individual due to the sharing of susceptibility genes. Both conditions have significant long-term health complications. Studies have shown some beneficial effects of a GFD on symptoms associated for patients with both conditions. The dietary management of both these conditions is challenging for the patient and requires support from specialist health professionals with specific focus on psychosocial and dietary support in CD and T1DM.
